# A Testis-Specific *DMRT1* (Double Sex and Mab-3-Related Transcription Factor 1) Plays a Role in Spermatogenesis and Gonadal Development in the Hermaphrodite Boring Giant Clam *Tridacna crocea*

**DOI:** 10.3390/ijms25115574

**Published:** 2024-05-21

**Authors:** Zohaib Noor, Zhen Zhao, Shuming Guo, Zonglu Wei, Borui Cai, Yanping Qin, Haitao Ma, Ziniu Yu, Jun Li, Yuehuan Zhang

**Affiliations:** 1 Key Laboratory of Tropical Marine Bio-Resources and Ecology, Guangdong Provincial Key Laboratory of Applied Marine Biology, South China Sea Institute of Oceanology, Chinese Academy of Sciences, Guangzhou 510301, China; zohaibnoor@mails.ucas.ac.cn (Z.N.); zhaozhen1995@petalmail.com (Z.Z.); guoshuming21@mails.ucas.ac.cn (S.G.); weizonglu@yeah.net (Z.W.); cb828@foxmail.com (B.C.); qinyanping@scsio.ac.cn (Y.Q.); htma@scsio.ac.cn (H.M.); zl15920537852@163.com (Z.Y.); 2 University of Chinese Academy of Sciences, Beijing 100049, China; 3 Southern Marine Science and Engineering Guangdong Laboratory (Zhuhai), Zhuhai 519015, China; 4 Hainan Provincial Key Laboratory of Tropical Marine Biology Technology, Sanya Institute of Oceanology Chinese Academy of Sciences, Sanya 572024, China; 5 Animal Science and Technology College, Guangxi University, Nanning 530004, China

**Keywords:** *DMRT1*, spermatogenesis, *Tridacna crocea*, male-biased gene, hermaphrodite

## Abstract

The testis-specific double sex and mab-3-related transcription factor 1 (*DMRT1*) has long been recognized as a crucial player in sex determination across vertebrates, and its essential role in gonadal development and the regulation of spermatogenesis is well established. Here, we report the cloning of the key spermatogenesis-related *DMRT1* cDNA, named *Tc-DMRT1*, from the gonads of *Tridacna crocea* (*T. crocea*), with a molecular weight of 41.93 kDa and an isoelectric point of 7.83 (pI). Our hypothesis is that *DMRT1* machinery governs spermatogenesis and regulates gonadogenesis. RNAi-mediated *Tc-DMRT1* knockdown revealed its critical role in hindering spermatogenesis and reducing expression levels in boring giant clams. A histological analysis showed structural changes, with normal sperm cell counts in the control group (ds-EGFP) but significantly lower concentrations of sperm cells in the experimental group (*ds-DMRT1*). *DMRT1* transcripts during embryogenesis exhibited a significantly high expression pattern (*p* < 0.05) during the early zygote stage, and whole-embryo in-situ hybridization confirmed its expression pattern throughout embryogenesis. A qRT-PCR analysis of various reproductive stages revealed an abundant expression of *Tc-DMRT1* in the gonads during the male reproductive stage. In-situ hybridization showed tissue-specific expression of *DMRT1*, with a positive signal detected in male-stage gonadal tissues comprising sperm cells, while no signal was detected in other stages. Our study findings provide an initial understanding of the *DMRT1* molecular machinery controlling spermatogenesis and its specificity in male-stage gonads of the key bivalve species, *Tridacna crocea*, and suggest that *DMRT1* predominantly functions as a key regulator of spermatogenesis in giant clams.

## 1. Introduction

Giant clams are widely distributed in the tropical marine waters of the Indo-Pacific region, with prominent species identified in the South China Sea, living at a depth of approximately 20 m embedded in shallow coral reefs. Only thirteen species of giant clams are known today, with only eight species identified in the South China Sea. *Tridacna* plays a crucial biological role in preserving and sustaining coral reef ecosystems [1,2]. They are considered synchronized bisexuals, with both male and female gonads present in the same animal and same organ, with male gonads maturing first and female gonads maturing later. During the spawning season, they typically release sperm first, followed by eggs after a brief period, and both gametes fertilize outside the body [3]. In recent decades, due to the over-exploitation of clam resources and habitat loss caused by human activities, giant clam populations have been highly threatened throughout their geographic ranges [4]. While efforts have been made to restock and induce breeding, which may help alleviate the situation to some degree, the molecular machinery controlling gonadal development needs further evaluation [5]. To genuinely support restoration efforts, it is essential to understand the fundamental molecular pathways that govern gonadal development and gametogenesis.

It has been investigated that, in the majority of animal species, sex determination and differentiation are commonly assisted by signal transduction cascades. In the fruit fly (*Drosophila melanogaster*), the signal transduction cascade was studied as the sex-lethal (Sxl)-transformer (Sxl)-transformer (Affairs)-doublesex (Dsx) pathway, controlled by a set of genes [6]. The sex-determining cascade in the nematode *Caenorhabditis elegans* consists of an ordered series of regulatory genes, including Xol-1, Her-1, Fem-1,2,3, and Tra-1,2,3 [7]. The assigned genes ensure gonadal development, differentiation, and gametogenesis. In decapod crustaceans, the development of male sex characteristics is regulated by the insulin-like androgenic gland hormone (IAG), which is thought to be produced and secreted by a male-specific endocrine organ, the androgenic gland (AG) [8,9,10,11]. Furthermore, mollusks, particularly bivalves, have developed diverse reproductive strategies, including dioecy and hermaphroditism, and some species exhibit sex reversal capabilities. However, a significant knowledge gap remains in understanding the precise molecular pathways involved in sex determination in mollusk species. Notably, the *DMRT1* gene, which is highly conserved across various animal taxa, plays a crucial role in regulating testis differentiation and development [12,13,14,15]. In Medaka (*Oryzias latipes*), the discovery of *DMRT* reveals a male sex-determining gene called dmY/dmrtY, which is considered functionally parallel to the mammalian SRY. *DMRT* family genes are highly conserved in vertebrate species and are considered to be vertebrate transcription factors, comprising a DNA motif (DM domain). In invertebrates, the doublesex (dsx) DNA-binding motif is also conserved, including in the nematode male-biased gene, male abnormal 3 (mab-3). Based on the outcomes of previous studies, it is confirmed that the functions of DM domain protein in sex determination are conserved and active within a wide scope of metazoans [16,17,18,19].

It is believed that among *DMRT* genes, only *DMRT1* is expressed in the gonad, which regulates gametogenesis, gonadal differentiation, and sex determination in various vertebrates [20,21,22,23]. Furthermore, all members of the *DMRT* family genes control sex determination in numerous vertebrate phyla [14,22,24]. For instance, in one or both sexes of the mouse fetal gonad, several *DMRT* genes are expressed, and it has been observed that solely *DMRT1* is involved in gonadal function before birth [25]. The *DMRT1* protein is expressed and becomes male-specific by approximately E14.5, when ovarian and testicular structures are examined in mice [21,26,27,28]. These findings suggest that genes for sex differentiation and determination are highly conserved among various phyla. In reproduction, sex determination is a fundamental and crucial process for the survival and evolution of vertebrate species. However, for bivalve mollusks, especially giant clams, there are very little data on the validation of *DMRT1* gene-related functions. Therefore, it is particularly important to pursue a comprehensive understanding of the role of *DMRT1* in gonadal development and spermatogenesis in giant clams.

In this study, we successfully cloned the full-length sequence of the *Tc-DMRT1* gene from the giant clam gonad. To evaluate the functional attributes of *DMRT1* in the giant clam, we conducted an RNAi experiment. We explored the tissue-specific expression of *Tc-DMRT1* in different reproductive stages using in-situ hybridization and qRT-PCR. Additionally, we examined the expression pattern of *Tc-DMRT1* in various embryonic stages using whole-embryo in-situ hybridization. Our research provides valuable insights into the reproductive biology of giant clams, shedding light on key processes, such as spermatogenesis, gonadal development, and maternal gene expression. These findings offer important theoretical references for future breeding programs and conservation efforts.

## 2. Results

### 2.1. Nucleotides, Translated Amino Acid Profile, and Phylogeny of Tc-DMRT1 Gene

**Figure 1:**
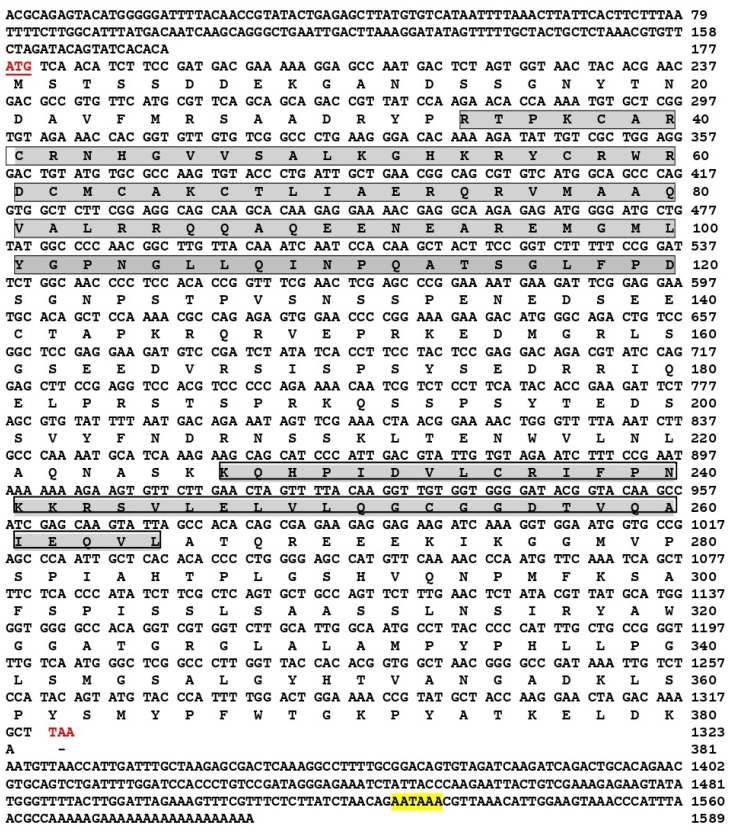
The full-length nucleotide sequence of *T. crocea DMRT1* cDNA and the predicted protein sequence are shown below. The start and stop codons are highlighted with red and bold. The DM and CUE-like DMA domain are represented in the enclosed box pattern. The tail signal is highlighted yellow.

**Figure 2:**
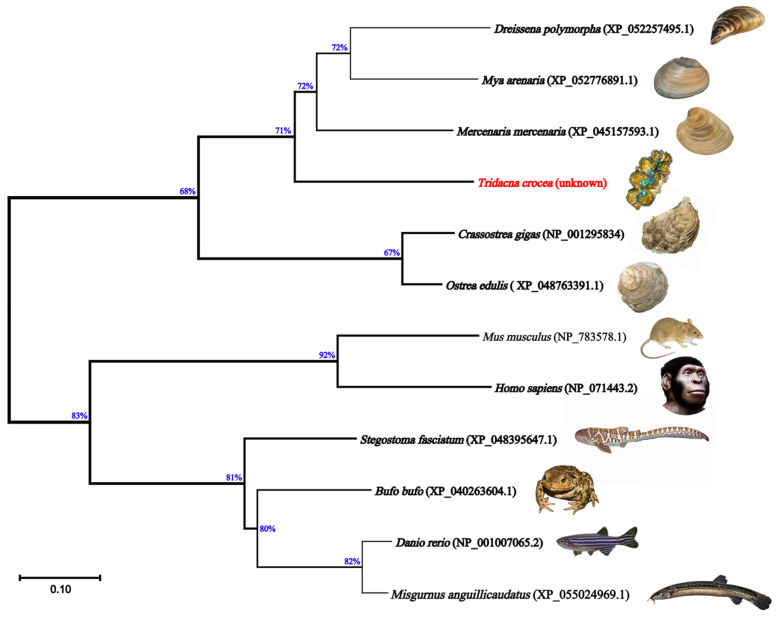
A neighbor-joining tree of *Tc-DMRT1* was constructed for comparison with vertebrates and bivalve species based on amino acid sequences from *Mercenaria mercenaria* (XP_045157593.1), *Crassostrea gigas* (NP001295834), *Dreissena polymorpha*, (XP_052257495.1), *Ostrea edilus* (XP_048763391.1), *Mya arenaria* (XP_052776891.1), *Danio rerio* (NP001007065.2), *Homo sapiens* (NP 1154486.1), *Mus musculus* (NP_783578.1), *Bufo bufo* (XP_040263604.1), *Misguruns anguillicaudatus* (XP_055024969.1), and *Stegostoma fasciatum* (XP_048395647.1). The tree includes bootstrap values (1000 replicates) indicating the credibility of each clade. Valves denote bootstrap scores, providing a measure of the reliability of each branch.

**Figure 3:**
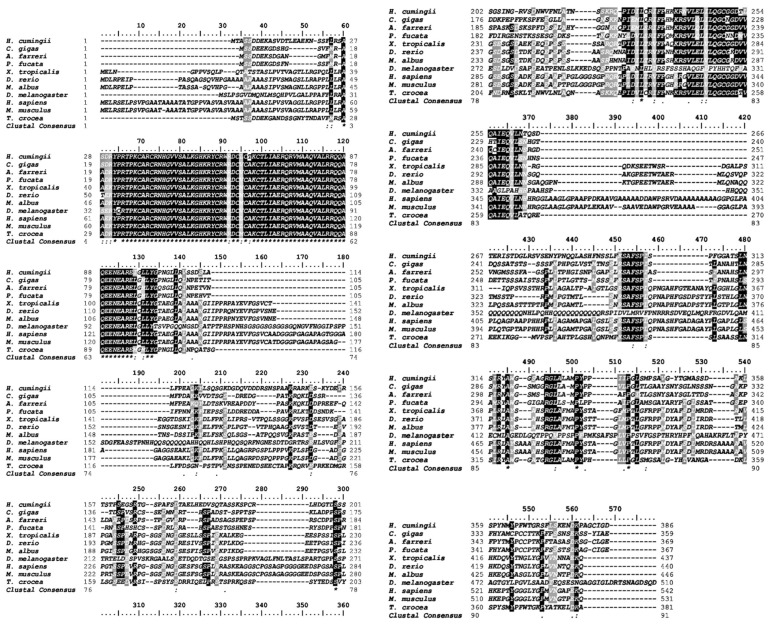
Multiple sequence alignments of the putative amino acid sequence of *Tc-DMRT1* cloned from *Tridacna crocea*, aligned with the sequences from other animals and bivalve species. The key DM domain with the highest likeness is highlighted grey and indicated in the box. Asterisk symbols correspond to 100%, two dots to 80%, and one dot to 60% similarity, respectively. Gene accession numbers are given as *Crassostrea gigas* (NP_001295834), *Danio rerio* (NP_001007065.2), *Drosophila melanogaster* (NP_524549.1), *Homo sapiens* (NP 1154486.1), *Mus musculus* (NP758500.2), and *Tridacna crocea* (unknown).

The full-length sequence of the *Tc-DMRT1* gene was retrieved from the gonads of *T. crocea*. The 5′-untranslated region (5′-UTR) consists of 178 bp, while the 3′-UTR consists of 269 bp. The open reading frame (ORF) sequence of 1146 bp encodes a polypeptide of 381 amino acid residues, with a predicted molecular weight of approximately 41.93 kDa. The full-length gene spans 1589 bp, including UTRs obtained from *T. crocea* gonadal tissues through PCR amplification. A polyadenylation signal (AATAAA) is located 39 bp upstream of the poly-A tail of *Tc-DMRT1*. The putative amino acid sequence contains the conserved DM domain and CUE-like DMA (Figure 1). Phylogenetic analysis of the deduced amino acid sequence of *Tc-DMRT1* revealed two major clades: one comprising vertebrates, including *Danio rerio*, *Bufo bufo*, *Mus musculus*, and *Stegostoma fasciatum DMRT1* sequences, and another comprising invertebrates, including *Crassostrea gigas*, *Pinctada fucata*, and *Dreissena polymorpha*. *T. crocea* clustered in the bivalve clade with *Mercenaria mercenaria* (Figure 2). Multiple sequence alignment revealed the conserved DM domain in *Tc-DMRT1*, which shows complete conservation with other species, including higher vertebrates, and shares the highest sequence similarity with its closely related mollusk species (Figure 3). These results indicate the conserved status of *Tc-DMRT1* in giant clams and rest of the animals in the clades.

### 2.2. Expression Pattern of Tc-DMRTI during Various Reproductive Stages

**Figure 4:**
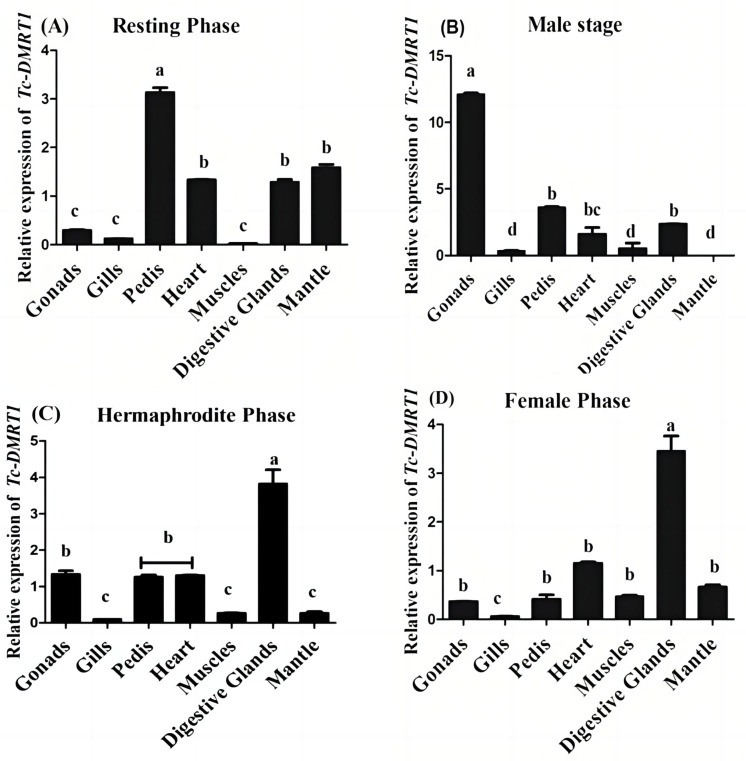
The transcript level of *Tc-DMRT1* is represented as mean ± SD (N = 5) in the bar graph. The different reproductive stages of giant clams are shown in the montage pictures. (**A**) Resting phase: the absence of male and female gametes in gonadal tissues. (**B**) Male stage: the presence of male gametes (sperm) Only. (**C**) Hermaphrodite stage: the presence of both sperm and ova. (**D**) Female phase: the presence of ova only. The different alphabetical symbols (a, b, c, and d) represent significant differences between various tissues (*p* < 0.05).

The expression pattern of *Tc-DMRT1* in giant clam gonads was evaluated across various stages, including resting, male, hermaphrodite, and female stage. Quantitative PCR analysis revealed that *Tc-DMRT1* expression is significantly up-regulated during the resting stage in the pedis, followed by lower expression in the heart tissues. However, expression remains relatively low in the gonads, gills, and muscles. Notably, during the hermaphroditic stage, the gene shows significant up-regulation in the digestive glands (*p* < 0.05), while expression remains low in the gills, pedis, heart, muscles, gonads, and mantle tissues. In contrast, the male stage exhibits an exacerbated expression level in the gonads, with the highest overall expression observed. Meanwhile, the female stage shows elevated expression levels in the digestive tract (*p* < 0.05), with negligible concentrations of *Tc-DMRT1* transcript observed in the ctenidium (Figure 4). These results suggest subtle differences in *Tc-DMRT1* expression across various stages and organs, with the male stage of gonads exhibiting the highest overall expression.

### 2.3. In-Situ Hybridization for the Localization of Tc-DMRT1 mRNA during Various Reproductive Stages

**Figure 5:**
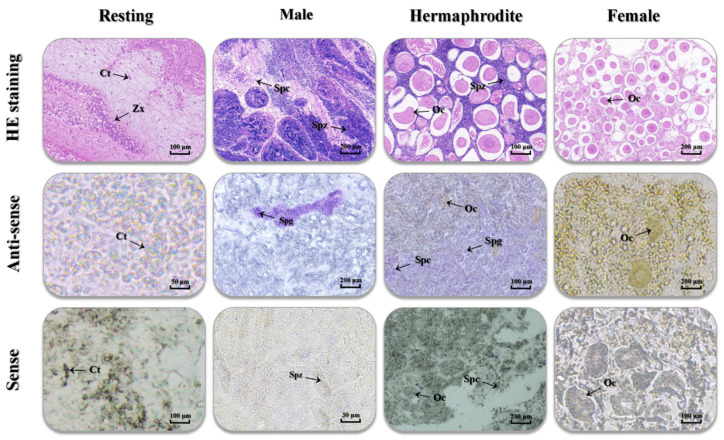
Localization of a specific segment of *Tc-DMRT1* was achieved using an antisense probe within histological sections of gonads at different stages. The figure shows the gonadal histology sections of the male stage (sperm cells), female stage (egg cells), and resting stage (connective tissues). Tissue sections of gonads treated with an antisense *Tc-DMRT1* probe and a sense probe (control). Various reproductive stages of *Tc-DMRT1* expression in spermatocytes (SPCs), spermatogonia (SPG), spermatozoa (SPZ), oocytes (OCs), and connective tissues (CTs).

Tissues from the male stage, which contain sperm cells, primarily spermatogonia, exhibited positive signals when probed with the antisense probe, confirming the expression of *Tc-DMRT1* in sperm cells. In contrast, no signal was detected when using the sense probe, serving as a negative control. Repeated in-situ hybridization (ISH) experiments using the antisense probe on female stage tissues, comprising oocyte cells, failed to show a positive signal, indicating the absence of *Tc-DMRT1* expression in oocyte cells of the gonadal tissues. Similarly, no cross-hybridization signal was observed in the resting stage when using both sense and antisense probes, confirming the absence of *Tc-DMRT1* expression in the connective tissue of the gonads. Therefore, ISH experiments demonstrated that *Tc-DMRT1* is exclusively expressed in the sperm cells of giant clams, supporting its role in male reproduction (Figure 5).

### 2.4. ds-DMRT1 Injection

**Figure 6:**
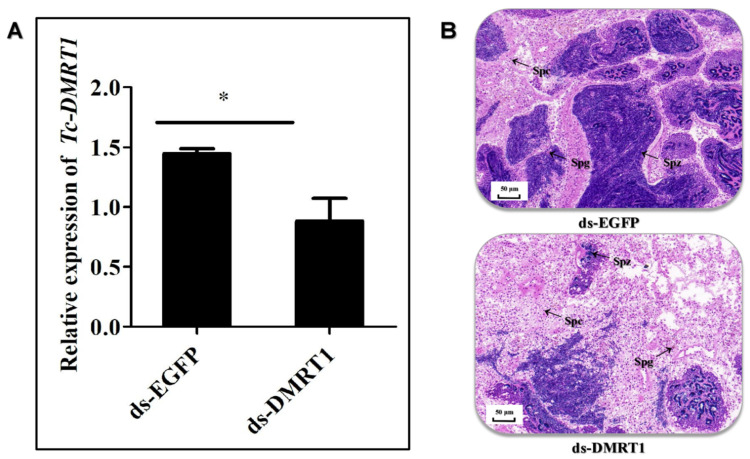
RNAi knockdown of *Tc- DMRT1*. (**A**) *Tc-DMRT1* expression pattern after ds-*DMRT1* injection. The post-injection profile of *Tc-DMRT1* was analyzed by qPCR in gonadal tissues, showing a significant reduction in *Tc-DMRT1* expression. The bar graph represents mean ± SD (N = 5), with a significant difference between *Tc-DMRT1* and ds-GFP (* *p* < 0.05). (**B**) Histological attributes of gonadal tissues of giant clams after ds-RNA injections, showing changes in gonadal tissue.

Fourteen days after administering dsRNA injections, we quantified the mRNA transcripts of *Tc-DMRT1* in giant clams using qRT-PCR. The results showed significant down-regulation of *Tc-DMRT1* expression (*p* < 0.05) in the experimental group receiving in-vivo ds-*DMRT1* injections, validating the inhibitory effect of RNA interference. In contrast, the control group (EGFP) showed an upstream expression profile of the *Tc-DMRT1* transcript. The injection of dsRNA into giant clams resulted in the blocking of *Tc-DMRT1*, leading to reduced spermatogenesis and a significantly lower number of sperm cells in the gonadal tissues of the experimental group (Figure 6A). Histological sections from the EGFP group showed a large aggregation of sperm cells in the middle and outer ridges, whereas the ds-*DMRT1*-treated group had fewer sperm cells in the outer ridges, indicating a clear histological differentiation between the two groups (Figure 6B).

### 2.5. Spatial and Temporal Expression Pattern of Tc-DMRT1 during Embryogenesis

**Figure 7:**
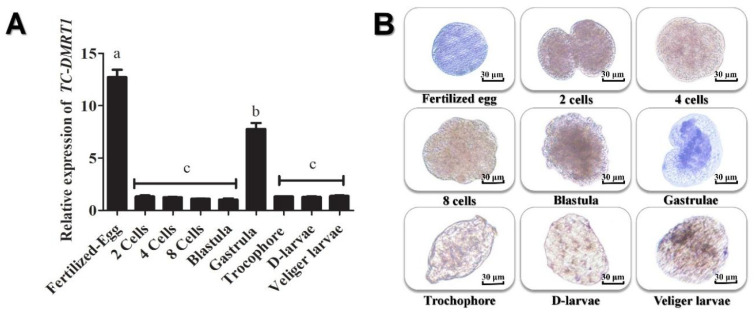
Expression trend of *Tc-DMRT1* in various embryonic stages of *Tridacna crocea*. (**A**) Comparative analysis of *Tc-DMRT1* transcript levels during different embryological stages. Results represent means ± SEM (n = 5) in the bar graphs, with a significance level of *p* < 0.05.The different alphabetical symbols (a, b, and c) represent significant differences between various tissues (*p* < 0.05). (**B**) A whole-embryo in-situ hybridization technique was used to localize the nucleotide sequence of *Tc-DMRT1* during early embryonic stages. Antisense probe-treated samples were visualized with an NBT solution, appearing blue/purple. The figure shows various embryonic stages, including a fertilized egg, 2-cell stage, 4-cell stage, 8-cell stage, blastula, gastrula, Trochophore larvae, D-shaped larvae, and Veliger larvae. This figure demonstrates the spatial and temporal expression pattern of *Tc-DMRT1* during *Tridacna crocea* embryonic development.

To investigate the *DMRT1* mRNA level during early embryo development and metamorphosis in giant clams, we analyzed different developmental stages for differential transcript levels. The results revealed a peak in *DMRT1* expression during early embryogenesis, followed by a gradual decline in 2, 4, and 8-cells blastomeres. However, *DMRT1* expression increased during gastrulation and then gradually decreased during the D-larvae, trochophore, and juvenile stages (Figure 7A). To further examine *DMRT1* expression during embryogenesis, we employed whole-embryo in-situ hybridization using an antisense probe to detect the mRNA nucleotides of the *Tc-DMRT1* gene in various embryonic stages. The fertilized egg showed a strong positive signal with the antisense probe, followed by faint signals in different cleavage stages. The blastula exhibited weak signals, while strong expression was observed in the gastrula, and the positive signal remained stable until the veliger stage (Figure 7B).

## 3. Discussion

The role of *DMRT1* orthologs has long been deemed crucial in sex determination, gonad development, and maintenance throughout evolutionary history. It acts as a functional regulator of mammalian gonads and as a differentiation and determination factor for sex, and engages in the normal progression of spermatogenesis in major phyla. *DMRT1* is imperative in Sertoli cells to prevent female reprogramming of the adult animal’s testes [29] and is requisite in spermatogonia to maintain their proliferative phase by evading meiotic entry [30]. Another study has shown that *DMRT1* presumably acts as a bifunctional transcriptional regulator by activating certain pivotal genes necessary for the absolute development of the male phenotype, while blocking the expression of others responsible for female gonad differentiation [31]. These studies register the pivotal role of *Tc-DMRT1* in the spermatogenesis of boring giant clams.

Elucidating the regulatory role of *Tc-DMRT1* during spermatogenesis would be fascinating, as its dynamics likely contribute significantly to the phenotype. Notably, this study is the first to explore the functional properties of *Tc-DMRT1* in giant clams. The full-length sequence obtained was 1589 bp, with an open reading frame (ORF) length of 1146 bp. The phylogenetic analysis revealed that the DM region is a conserved sequence among vertebrates, including humans, zebrafish, and mice, while the *Tc-DMRT1* protein clusters closely with its mollusk analogs. The conserved DM domain comprises cysteine residues, which likely form a zinc-finger motif. Similarly, a study on Japanese eel fish *DMRT1* found a conserved DM domain, zinc finger motif, and six conserved cysteine residues [32]. *Haliotis asinine,* a common marine mollusk, has a complete *DMRT1* length of 1740 bp, with a predicted ORF range of 732 bp, translating to 243 amino acids. Multiple sequence alignment confirmed the conserved status of the regulatory DM domain in the giant clam (Figure 1).

A transcriptome analysis of *Pinctada margaritifera* revealed the presence of testis-specific genes encoding proteins involved in sex determination and gonad differentiation, such as fem-1-like and *DMRT1*, which are exclusively expressed in male testes [33]. This finding supports our conclusion regarding the testis-specific expression of *DMRT1* and its crucial role in sex determination. Furthermore, a comparative analysis of giant clams identified candidate genes responsible for gametogenesis, with *DMRT1* emerging as a testis-specific gene potentially involved in male gonadal development (Figure 4) [23].

The expression level of *DMRT1* remains high during spermatogenesis in rainbow trout, followed by a significant decrease during spermiation. However, its expression remains low during gonadogenesis [34]. Previous studies have shown that *DMRT1* expression is exclusively restricted to the testes and varies according to different spermatogenetic phases in pejerrey fish (*Odontesthes bonariensis*). Fernandino et al. (2006) confirmed that *DMRT1* expression is crucial during the initiation and maintenance stages of spermatogenesis. Similarly, in air-breathing catfish, *DMRT1* concentrations remain dormant during post-spawning stages. The sustained expression of *DMRT1* throughout spermatogenesis leads to the down-regulation of SoxE and another ovarian regulator, foxl2 [35]. These studies highlight the positive role of *DMRT1* as a co-regulator of SoxE and reinforce its key role in testicular development. Furthermore, RNAi injection in giant clams resulted in the down-regulation of *Tc-DMRT1*, confirmed by testis histology, which showed significantly lower sperm cell counts in the RNAi-injected group compared to the EGFP group, which had prominent sperm counts [36]. The RNAi experiment endorses the functional role of *Tc-DMRT1* in giant clams, and the low sperm counts suggest that *Tc-DMRT1* knockdown impedes spermatogenesis (Figure 6A).

In the gonads of *Pinctada margaritifera*, the male-specific gene *DMRT1* shows an increasing trend throughout spermatogenesis, from early development to mature phases [33]. Similarly, *DMRT1* is exclusively expressed in the testis of *Haliotis asinine* [37]. We observed high amplification trends of *DMRT1* only in the testes, with a lower trend in hemocytes. The tissue-specific expression in *Haliotis asinine* gonads independently confirms its exclusive expression in gonadal tissues, suggesting its role in testicular development. In vertebrates, such as chickens, elevated *DMRT1* concentrations have been detected in female-to-male sex-reversed embryos [38]. The hermaphroditic fish species *Epinephelus coioides*, which has gonads that mature as ovaries before reverting to the testes, also expresses *DMRT1* during spermatogenesis [39]. A Northern blot analysis revealed testis-specific functions of *DMRT1* in protogynous wrasse fish, indicating its key role in sex determination and sex-related functions [40]. Other studies demonstrate *DMRT1* expression in both germ and somatic cells in animals like chickens [41], frog *Xenopus laevis*, [42], and mice [27]. Mouse *DMRT1* is suggested to play indispensable roles, including germ-cell development and somatic cell masculinization [30]. Although robust male-biased expression of *DMRT1* is a general trend, it has also been detected in the ovaries of various animal species. For instance, *DMRT1* expression was observed in spermatogonia of Atlantic cod, maturing oocytes, and immature ovary oogonia [43]. Similarly, in developing germ cells of zebrafish, *DMRT1* mRNA expression is detected in testes, developing oocytes, and various stages of oocyte development, including early peri-nucleolus stage oocytes, oil drop stage oocytes, and late yolk vesicle stage oocytes, as revealed by in-situ hybridization [44]. Another isoform of the *DMRT1* family, *DMRT3,* in *C. elegans*, is exclusively expressed in male gonads, where it promotes maleness [13,22,45]. Based on current findings, *Tc-DMRT1* can be assumed to be a testis-specific gene that likely enhances testis development and controls sperm development. While its expression may vary according to the developmental stage of giant clams, it is sustained during developmental stages, including fertilized eggs, 2-cells and 4-cells embryos, with a decreasing trend noticed post-gastrulation. Moreover, its testis-specific expression is confirmed by both qRT-PCR amplification and in-situ hybridization (ISH), collectively reaffirming its specificity in the testes.

## 4. Materials and Methods

### 4.1. Giant Clam Procurements and Maintenance Conditions

Adult *Tridacna crocea* species, with a wet mass of 451 ± 111 g, were obtained from the Tropical Marine Research Station in Sanya, Hainan Island, China. A total of 30 animals were used for the experiment, all of which were from the same generation and approximately three years old. The animals were maintained in indoor rearing tanks equipped with an aerated flow-through, sand-filtered water system, and all rearing conditions were artificially optimized to ensure their health and well-being [46]. They were maintained at a salinity of 20–30‰ and a temperature of 25.5–26.6 °C. Samples were collected in accordance with the guidelines set by the Guangzhou Animal Care and Use Committee, which granted an exemption from approval for the use of giant clams in this study.

### 4.2. Tissue Collection

After a brief 14-day acclimatization period, the giant clams were sampled for various experiments and analyses. For analytical purposes, seven targeted organs tissues were collected, including the mantle, heart, gonads, pedis, digestive glands, muscles, and ctenidium (gills) [47]. The animal shell was forcibly opened by invasive cutting of the adductor muscles to collect the samples. Additionally, different developmental stages, including fertilized eggs, 2-cells, 4-cells, 8-cells, blastula, gastrula, trochophore, D-larvae, and veliger larvae, were confirmed by microscopy and selected accordingly for further analysis. After removal, tissue samples were snap-frozen in liquid nitrogen and stored at −80 °C until further analysis [48].

### 4.3. Total RNA Extraction, cDNA Synthesis, PCR, Cloning, and RACE-PCR

Total RNA was extracted from various adult giant clam tissues using Triazole reagent (Invitrogen, Waltham, MA, USA) and then reverse transcribed into cDNA using the PrimeScript^TM^ RT reagent Kit (with gDNA Eraser) (TaKaRa, Kusatsu, Japan). A total of 2-5 µg of RNA was transcribed into 40 μL of cDNA. Fresh RNA samples isolated from giant clam gonads were pooled in triplicate and used as templates for RACE-PCR (Rapid Amplification of cDNA Ends Polymerase Chain Reaction) with the SMARTer RACE 5′/3′ Kit (TaKaRa, Japan).

Using the *T. crocea* transcriptome database, we retrieved the partial sequence of *Tc-DMRT1* and obtained the ORF (ORFfinder, NCBI, Bethesda, MD, USA) and 5′ and 3′ UTRs through PCR amplifications. Primer 5.0 software was used for primer design. All primers used for amplification are listed in Table 1. The unknown sequences at the 3′ and 5′ ends of *Tc-DMRT1* were retrieved using the following PCR protocol: initial denaturation at 97 °C for 3 min, followed by 35 cycles of amplification at 97 °C for 30 s, 60–55 °C for 30 s, and 72 °C for 1 min, and a final elongation at 72 °C for 10 min. The PCR product was then diluted and used as a template for a second PCR, following the manufacturer’s protocol: denaturation at 94 °C for 5 min, followed by 35 amplifications at 94 °C for 15 s, 55 °C for 30 s, and 72 °C for 1 min. Finally, the desired product was gel purified, ligated into the T-vector pMD19-(TaKaRa, Japan), transfected into α5-competent *E. coli* cells, and grown on an Luria Broth (LB) plate overnight. Clean colonies were grown in a LB solution and sent for sequencing.

### 4.4. Quantitative Polymerase Chain Reaction

A quantitative polymerase chain reaction was used for the expression of *DMRT1* in various tissues of *T. crocea*. I.e., the mantle, heart, gonads, pedis, digestive glands, muscles, and ctenidium (gills) using a set of primers listed in Table 1. The 96-well Light Cycler 480II system (Roche, Indianapolis, IN, USA) was used for the qPCR. Templates were diluted ten times before further analysis by qRT-PCR. The reaction was run on a 96-well thermal cycler in a light Cycler 480II system (Roche, USA); 20 μL PCR volume contained 1 μL of cDNA templates, 0.5 μL of forward and reverse primers (10 μM), 8 μL of PCR-grade water, and 10 μL of 2 × SYBR Green PCR Master Mix (Roche, USA). The following steps prescribed by the manufacturer were used for qRT-PCR: the denaturation process at 94 °C for 1 min; 40 cycles of amplification briefly for 15 s performed at 95 °C, 57 °C for a further 30 s, 72 °C for 20 s, and finally 1 min is required for signal collection at 85 °C for 15 s in every cycle. Each sample of experimental and control reactions (*qRPL5*) was performed in triplicates, and the relative expression level of *Tc-DMRT1* was calculated by adopting the 2^−ΔΔct^ method [49].

### 4.5. Synthesis of dsRNA

This study aimed to investigate the functional role of *DMRT1* in spermatogenesis and gonadal development. We designed and amplified a sequence from the ORF region of *Tc-DMRT1* as a dsRNA target site, which was confirmed by sequencing using the gene-specific primers ds-F and ds-R. We then incorporated T7 promoter sequence into the primers to synthesize ds-DMRT1. Two separate PCRs were run using sequence-specific primers and T7 promoter-incorporated sequence primers, and the target band was gel-purified and transcribed using the Promega RiboMAX^TM^ Express RNAi System. The transcription products were mixed in vitro and purified according to the manufacturer’s guidelines. As a control, ds-GFP was obtained similarly, with a 400 bp fragment of GFP initially amplified from pEGFP-N1 and then transcribed to obtain the product. An in-vivo experiment was conducted to evaluate the interference phenomenon by ds-DMRT1. Twelve adult giant clams were randomly divided into control and treated groups, receiving intramuscular injections of ds-DMRT1 and ds-EGFP into the adductor muscles. The experimental group received 50 μL dsRNA injections on day one, while the control group received an equal volume of ds-EGFP. On day seven, both groups received the same dsRNA concentrations injected into their adductor muscles. Finally, on day fourteen, the giant clams were dissected to obtain gonads, which were examined for potential effects of ds-DMRT1 on spermatogenesis, validated through qPCR and histopathology.

### 4.6. In-Situ Hybridization (ISH) and Whole-Mount In-Situ Hybridization (WISH)

This study aimed to confirm the stage-specific and tissue-specific expression of *DMRT1* in gonadal tissues. Freshly sampled giant clams’ gonads were fixed with 4% paraformaldehyde and incubated at 4 °C overnight. The following day, tissues were thoroughly washed thrice with PBS and dehydrated using a 30% sucrose solution. Tissue sections were placed in a cube-shaped box made of aluminum foil, then fixed in OCT compound (Optimal cutting temperature) and snap-frozen at −80 °C. The rest of the procedure was followed for tissue sections, as described by [48], while for the whole embryo, methods adopted by [50] were used with specific alterations. Finally, positive signals were observed under a microscope. The expression of *DMRT1* in various tissues is visualized by the blue/purple color, which indicates the presence of the specific nucleotide sequence. This is achieved through the activity of Nitrotetrazolium Blue chloride (NBT), which causes the specific cells to glow, thereby validating the expression of *DMRT1* in those tissues.

### 4.7. Histology Sections

Samples collected for the ds-RNA experiment and qPCR were also used for the histology analysis to visualize exact reproductive stages. Histology sections were prepared from various gonadal stages, selected based on germ-cell development, including the resting stage, male, hermaphrodite, and female stages. The samples were dehydrated using a series of ethanol concentrations and then embedded in paraffin. Thin sections (6 μm) were cut using a microtome, stained with hematoxylin-eosin, and examined under a microscope [48].

### 4.8. Data Analysis

The data obtained were compiled from biological triplicates, and the statistical analysis was performed using SPSS 18.0 and GraphPad Prism 5 to identify significant differences. All data are presented as means ± SEM. A one-way ANOVA was followed by post-hoc tests, and a significance level of *p* < 0.05 was chosen to determine statistically significant variations. A neighbor-joining tree was constructed using MEGA11, with node values representing the percent bootstrap confidence derived from 1000 replicates. This tree provides a visual representation of the evolutionary relationships among the sequences, with bootstrap values indicating the confidence level of each node.

## 5. Conclusions

The present study aimed to isolate the full-length *DMRT1* from the boring giant clam, which is thought to be functionally responsible for gonadal development and spermatogenesis in vertebrates. Based on its functional properties in vertebrates, we successfully isolated and characterized *Tc-DMRT1*, which shows functional similarities with its vertebrate species and a conserved domain structure. RNAi knockdown of *DMRT1* confirmed its functional role in spermatogenesis by blocking sperm production due to its lower expression level. Tissue specificity revealed by in-situ hybridization (ISH) showed its expression in the male stage, while whole-mount in-situ hybridization (WISH) experiments confirmed the maternal expression of *DMRT1*. This study’s outcomes highlighted the significance of *DMRT1* in giant clams, which is essential for spermatogenesis. This study provides molecular data for further evaluations of *DMRT1* and its role in gonadogenesis and gametogenesis.

## Figures and Tables

**Table 1:** Primers used for the amplification of various transcripts of *Tc-DMRT1*.

Primer	Sequence (5′-3′)	Purpose
*Dmrt1*_5′ RACE1	CCATCTCTCTTGCCTCGTTTTCCT	5′ RACE of *Tc-DMRT1*
*DMRT1*_5′ RACE2	GTCAGTGGCTCCTTTTTCGTCATC	
*DMRT1*_3′ RACE1	ATCTTCGCTCAGTGCTGCCAGTTCTC	3′ RACE of *Tc-DMRT1*
*DMRT1*_3′ RACE2	CTGGAAAACCGTATGCTACCAAGGA	
*qDMRT1* 5′	CTTGGCATTTATGACAATCAAGC	Quantification of *DMRT1*
*qDMRT1* 3′	ACGGCGTCGTTCGTGTAGTT	
*qRPL5*_5′	CATACTCACACGAACTGCCTC	Reference ribosomal protein L5 gene for reproductive stages
*qRPL5*_3′	CTCCGTCTACTTCTGTCTGTCC	
*DMRT1*_RNAi_5′	TCAGCAGCAGACCGTTATCCGGATCCTAATACGACTCACTATAGG	RNA interference
*DMRT1*_RNAi_3′	GGATCCTAATACGACTCACTATAGGTCGGCACCATTCCACCTTT	

## Data Availability

The data are only available on request and are saved in our repository.
